# Transient Creatine Kinase Elevation Followed by Hypocomplementemia in a Case of Rotavirus Myositis

**DOI:** 10.1155/2016/3034170

**Published:** 2016-03-13

**Authors:** Yuka Rokugo, Satoru Kumaki, Ryoichi Onuma, Rie Noguchi, Saeko Suzuki, Natsuko Kusaka, Yohei Watanabe, Setsuko Kitaoka

**Affiliations:** Department of Pediatrics, Sendai Medical Center, 2-8-8 Miyagino, Miyagino-ku, Sendai, Miyagi 983-8520, Japan

## Abstract

We report an infant case of rotavirus myositis, a rare complication of rotavirus infection. Complement levels of the patient were normal when serum creatine kinase (CK) level was at its peak and then decreased when the CK level became normalized. In a previous case report of rotavirus myositis, transient decrease of serum albumin, immunoglobulin, and complement levels was reported. The authors speculated that intravascular complement activation was caused by rotavirus and resulted in the pathogenesis of myositis, although complement levels at onset were not measured by the authors. In this report, however, we demonstrate that the complement activation of our patient is a result of, rather than the cause of, skeletal muscle damage.

## 1. Introduction

Rotavirus infection is a common cause of acute gastroenteritis among infants. Extraintestinal complications such as encephalitis and myositis are rare [1]. So far, only two cases of rotavirus myositis have been reported [2, 3]. Bonno et al. speculate that complement activation is a cause for rhabdomyolysis [3]. Previous studies using animal models, however, demonstrate that complements play an essential role for the recognition and clearance of dead muscle cells by phagocytic macrophages and are not involved in the pathogenesis of virus-induced myositis [4, 5]. Recently, we experienced the case of a patient with rotavirus myositis that supports the latter case.

## 2. Case Presentation

A previously healthy 3-year-old boy visited a clinic after 4-day history of watery diarrhea and vomiting. Rotavirus was detected from his stool, leading to a diagnosis of rotavirus gastroenteritis. He was referred to our hospital because he was drowsy and reluctant to move even after he had received saline intravenously. Upon admission to our hospital on day 4, he could not stand or walk. Laboratory data showed markedly elevated serum creatine kinase (CK) 11637 IU/L, with mildly elevated serum enzymes including lactate dehydrogenase 691 IU/L, alanine aminotransferase 117 IU/L, aspartate aminotransferase 415 IU/L, and aldolase 118.9 U/L (2.7–7.5). Myoglobin was also elevated to 380 ng/mL (20–82). Serum complement levels were all normal: C3 83 mg/dL, C4 22 mg/dL, and CH50 33.9 U/mL. Other laboratory data were normal except for glucose 56 mg/dL, uric acid 9.4 mg/dL, C-reactive protein 1.5 mg/dL, and soluble interleukin-2 receptor (sIL-2R) 979.5 U/mL (332.9–586.7). Occult blood was not detected in the urine. Stool bacterial culture and throat bacterial and viral cultures were all negative.

**Figure 1:**
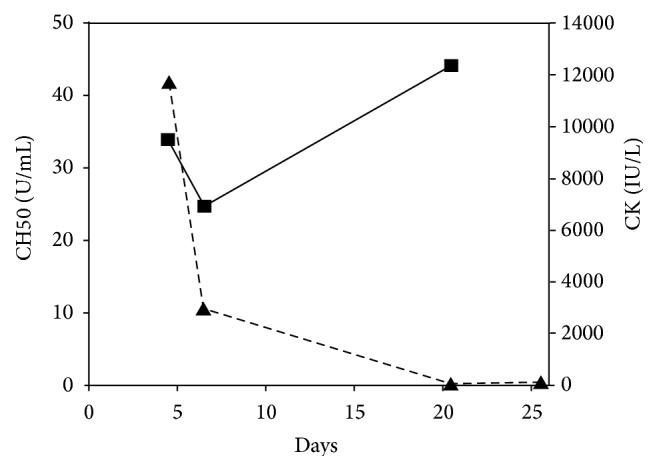
Clinical course of creatine kinase (CK) and CH50. The values of CK (▲) and CH50 (■) are indicated in the right and left axes, respectively.

With a diagnosis of rotavirus gastroenteritis and hypoglycemia, the patient was treated with intravenous glucose infusion and fluid therapy. Even after the serum glucose level was corrected, the patient was still reluctant to move. On day 6, his vomiting and diarrhea stopped. He could stand and walk by himself, although his movements were still unstable. On that day, his CK level rapidly returned to 2927 IU/L (Figure 1). In contrast, his serum CH50 activity decreased to 24.6 U/mL, with C3 75 mg/dL and C4 14 mg/dL. Other laboratory data were CRP 0.7 mg/dL and sIL-2R 1458.6 U/mL. He was discharged from our hospital without sequelae on day 9. Laboratory data were all normalized on day 21 and remained within the normal range thereafter.

## 3. Discussion

Only two cases of rotavirus myositis have been reported in the literature [2, 3]. One of them describes transient decrease of serum albumin, immunoglobulin, and complement levels on day 6 of the onset. The authors speculate that intravascular complement activation caused by rotavirus resulted in the pathogenesis of myositis, although complement titers were not measured by the authors at onset [3]. In our case, however, complement levels were normal when serum CK level was at its peak and decreased when CK level became normalized. Thus, we did not observe direct correlation between complement levels and CK level. Previous studies with animal models have demonstrated that complements are recruited to damaged tissues, playing a crucial role in facilitating phagocytic macrophages to recognize and clear dead cells during skeletal muscle regeneration [4, 5]. We therefore argue that the complement activation of our patient is a result of, rather than the cause of, skeletal muscle damage.

The mechanism of rotavirus myositis is not clear. In our case, a T cell activation marker sIL-2R increased along with complement activation but it showed negative correlation with both CK and CRP until day 6 of onset. In viral myositis, it was suggested that viruses initially trigger the disease process in muscle tissues and subsequently provoke immune responses [6]. Our result supports this hypothesis. In addition, we demonstrate that complement activation is a result of skeletal muscle damage. Further accumulation of cases will clarify the pathogenesis of rotavirus myositis.
